# Hidden in Plain Sight: Multifocal Asymptomatic Small Bowel Neuroendocrine Tumor Discovered During Robotic Excision of Incidentally Found Mesenteric Mass

**DOI:** 10.7759/cureus.111325

**Published:** 2026-06-22

**Authors:** Segala F Tita, Georgia Syrnioti, Leoga Tita, Jamal Zahir, Charles Thompson III

**Affiliations:** 1 Department of Surgery, Ross University School of Medicine, Miramar, USA; 2 Department of Surgery, Brookdale University Hospital Medical Center, Brooklyn, USA; 3 Department of General Surgery, Brookdale University Hospital Medical Center, Brooklyn, USA; 4 Department of Emergency Medicine, Ross University School of Medicine, Miramar, USA

**Keywords:** carcinoid tumor, gross abdominal anatomy, mesenteric lymphadenopathy, mesenteric mass, neuroendocrine tumor

## Abstract

Neuroendocrine tumors (NETs) of the small bowel are uncommon, typically slow-growing neoplasms that may remain asymptomatic until reaching advanced stages. We present the case of a 69-year-old male with multiple comorbidities who was referred for evaluation of an incidentally identified mesenteric lesion on computed tomography (CT). Preoperative imaging revealed a 5 × 3.6 × 4.3 cm node-like mass within the mesentery. During robotic exploration, this lesion was identified along with two additional previously undetected small-bowel NETs. The patient underwent small bowel resection with en bloc removal of the mesenteric mass, and his postoperative course was uneventful. Final pathology demonstrated multifocal, well-differentiated NET with 9 of 20 lymph nodes positive for metastatic involvement.

This case emphasizes important diagnostic and therapeutic considerations in the management of small-bowel NETs. Cross-sectional imaging can underestimate the true extent of disease, as multifocal tumors are common and may go undetected preoperatively. Complete surgical resection with negative margins remains the primary treatment modality and provides the best opportunity for long-term disease control. Minimally invasive and robotic approaches facilitate comprehensive intraoperative evaluation while preserving bowel length and vascular integrity. Early identification and definitive resection are critical, as untreated NETs can progress to complications such as mesenteric fibrosis, bowel obstruction, or metastatic spread despite their typically indolent course. This case highlights the importance of maintaining a high index of suspicion and performing careful intraoperative assessment when evaluating seemingly isolated mesenteric masses, given the potential for underlying multifocal disease.

## Introduction

Neuroendocrine tumors (NETs) of the small bowel are the most common primary malignancy of the small intestine, arising from enterochromaffin cells and typically exhibiting indolent growth but significant metastatic potential. The incidence of small bowel NETs has increased substantially over recent decades, a trend attributed to improved imaging modalities, endoscopic techniques, and heightened clinical awareness. Most NETs are sporadic, though a minority are associated with hereditary syndromes such as multiple endocrine neoplasia (MEN1, MEN2, MEN4), von Hippel-Lindau disease, and neurofibromatosis [1-8].

Clinically, small bowel NETs often present with nonspecific gastrointestinal symptoms, such as abdominal pain, diarrhea, or weight loss, or remain silent until advanced disease is reached. Carcinoid syndrome, characterized by episodic flushing, diarrhea, and valvular heart disease, occurs in a subset of patients, typically those with hepatic metastases, due to the systemic release of serotonin and other vasoactive substances. At diagnosis, a significant proportion of patients have regional lymph node or distant metastases, and mesenteric involvement is common, sometimes manifesting as fibrotic or calcified masses detected incidentally on cross-sectional imaging [1-2,4,5,7-15].

Diagnosis of small bowel NETs relies on a combination of radiologic, biochemical, and histopathologic evaluation. Multiphasic CT or MRI of the abdomen and pelvis is recommended for lesion detection, staging, and assessment of mesenteric involvement. Somatostatin receptor-based imaging offers improved sensitivity for tumor localization and staging and is particularly useful for identifying multifocal disease and guiding therapy. Small bowel endoscopy, including video capsule endoscopy and device-assisted enteroscopy, complements imaging by enabling direct visualization, localization, and biopsy of lesions that may be missed on radiology. Biochemical markers such as chromogranin A and 5-HIAA are useful for diagnosis and monitoring, especially in functional tumors [1-2,5,8-12,15,16].

Surgical resection remains the cornerstone of management for localized and select metastatic small bowel NETs, with complete excision of the primary tumor and regional lymphadenectomy recommended to prevent recurrence and alleviate complications such as obstruction or ischemia from mesenteric fibrosis. Intraoperative assessment should include manual palpation of the entire bowel to identify synchronous lesions and evaluation of proximity to the superior mesenteric vessels. For advanced or metastatic disease, multimodal therapy including somatostatin analogues, targeted agents, peptide receptor radionuclide therapy, and liver-directed interventions improves symptom control and progression-free survival. Long-term outcomes are favorable for many patients, with five-year survival rates exceeding 70% in population-based studies, though prognosis is influenced by stage, grade, and the presence of mesenteric tumor deposits [1-3,6-9,11-13].

Ongoing surveillance post-resection is recommended, with periodic imaging and biochemical assessment to detect recurrence or progression. The increasing incidence and complexity of small bowel NETs underscore the importance of multidisciplinary management, early diagnosis, and individualized treatment strategies to optimize patient outcomes [1-2,6,7,9,11,12].

The current World Health Organization (WHO) classification (2019, with subsequent updates) categorizes gastroenteropancreatic neuroendocrine neoplasms, including small bowel neuroendocrine tumors (SBNETs), based on both tumor differentiation and proliferative activity. Neoplasms are first divided into well-differentiated neuroendocrine tumors (NETs) and poorly differentiated neuroendocrine carcinomas (NECs), with an additional category of mixed neuroendocrine-non-neuroendocrine neoplasms (MiNENs). Well-differentiated NETs are graded using mitotic count and Ki-67 index into G1 (<3%), G2 (3-20%), and G3 (>20%), while NECs are, by definition, high-grade (G3) and include small- and large-cell subtypes. Importantly, the WHO 2019 update recognizes well-differentiated G3 NETs as a distinct entity from NECs despite sharing a Ki-67 >20%, reflecting differences in biology, prognosis, and management. In the small bowel, the majority of tumors are well-differentiated and fall within G1-G2 categories, with high-grade neoplasms being relatively uncommon [13].

## Case presentation

In this case, we have a 69-year-old obese male (BMI: 35 kg/m²) who was referred to the general surgery clinic for evaluation of an incidentally discovered mesenteric mass. The patient was asymptomatic and underwent a CT scan of the abdomen and pelvis for evaluation of an umbilical hernia. His past medical history is significant for hypertension, diabetes mellitus, congestive heart failure with an ejection fraction of 50%, and obstructive sleep apnea. He is a former smoker with a 50 pack-year smoking history and consumes alcohol socially. There was no family history of cancer.

**Figure 1:**
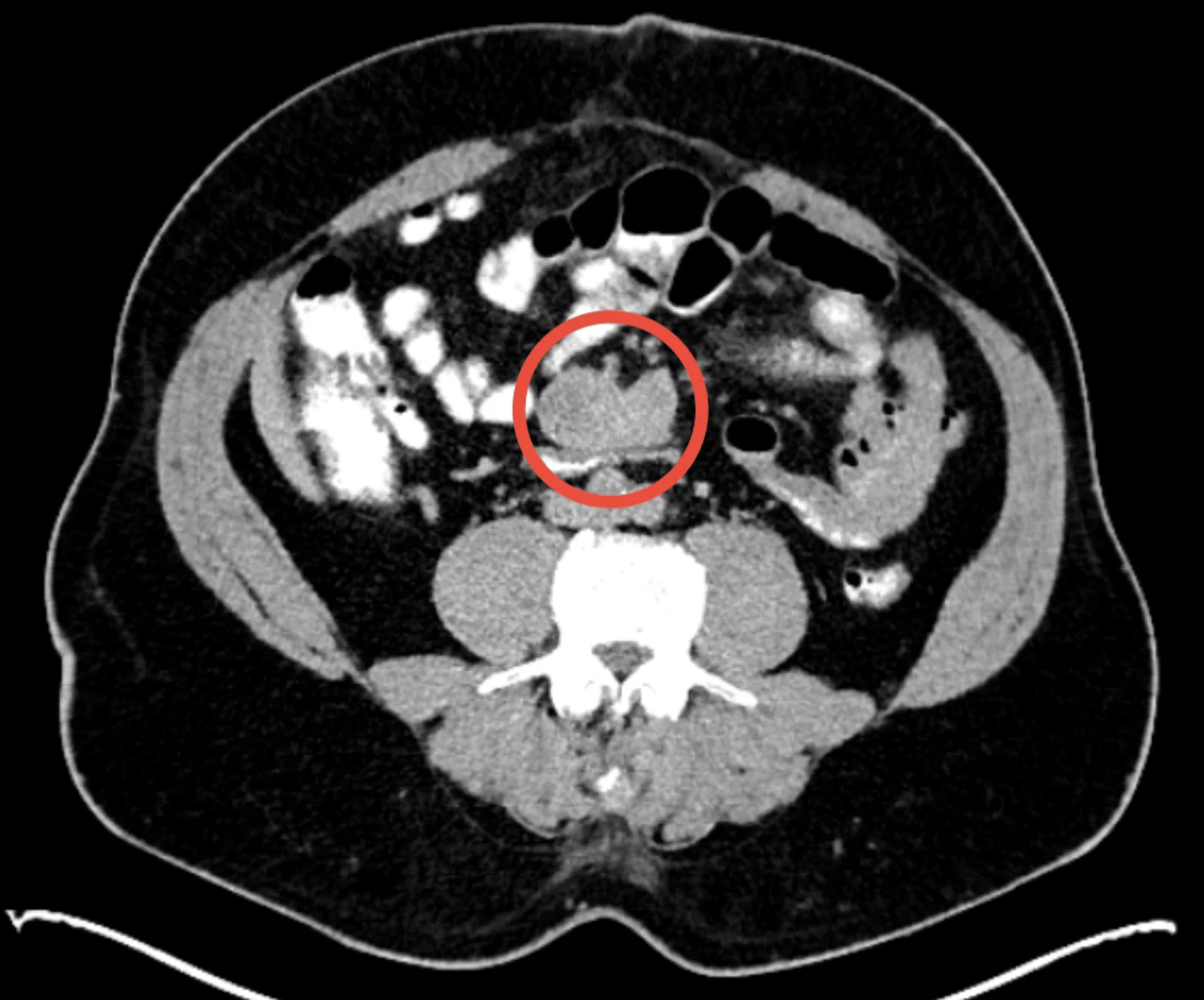
Axial view of a mesenteric mass measuring approximately 5 x 3.6 cm axially and 4.3 cm craniocaudally Red Circle: Mesenteric mass

**Figure 2:**
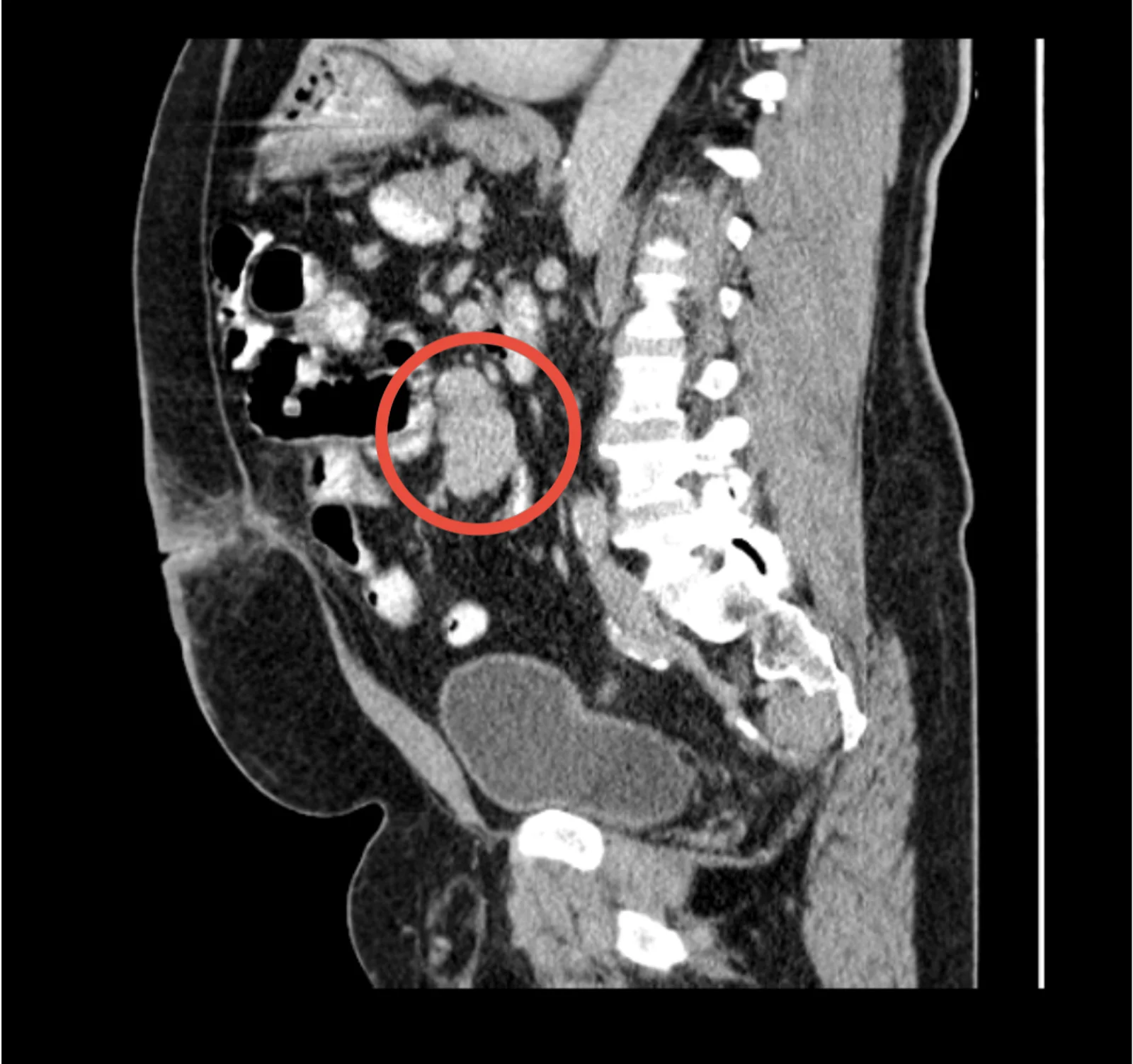
Sagittal view of a mesenteric mass measuring approximately 5 x 3.6 cm axially and 4.3 cm craniocaudally Red Circle: Mesenteric mass

Contrast-enhanced CT of the abdomen and pelvis revealed a markedly enlarged central mesenteric lymph node or cluster of lymph nodes measuring approximately 5 x 3.6 cm axially and 4.3 cm craniocaudally. No evidence of small bowel mass was noted on imaging. Figures 1, 2 demonstrate the presence of a mesenteric mass on the CT scan.

**Figure 3:**
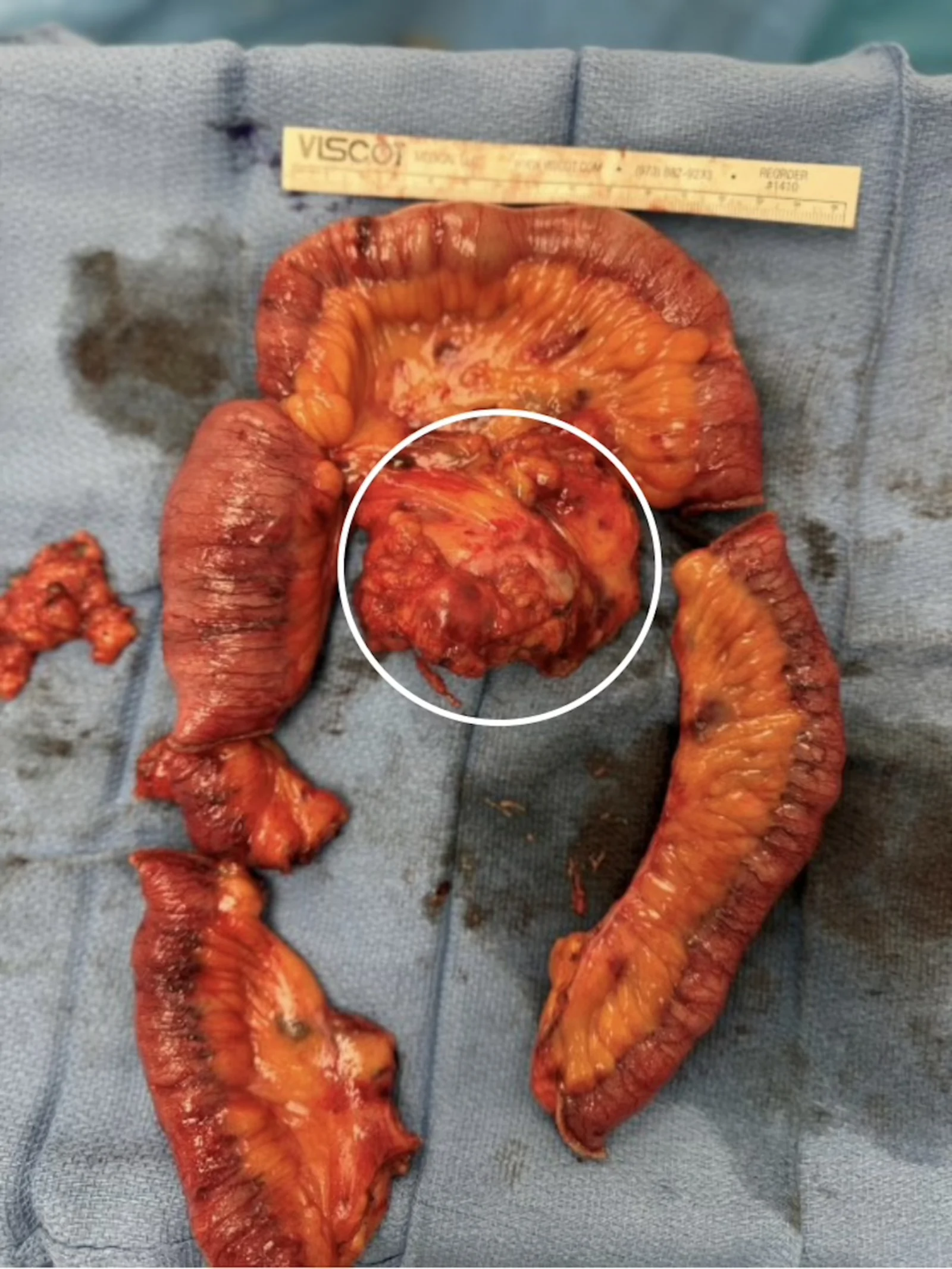
Gross pathologic specimen of the two small bowel masses with the associated mesenteric mass. This figure depicts the mesenteric mass along with 70 cm of jejunum that was resected The white circle demonstrates the mesenteric mass.

**Figure 4:**
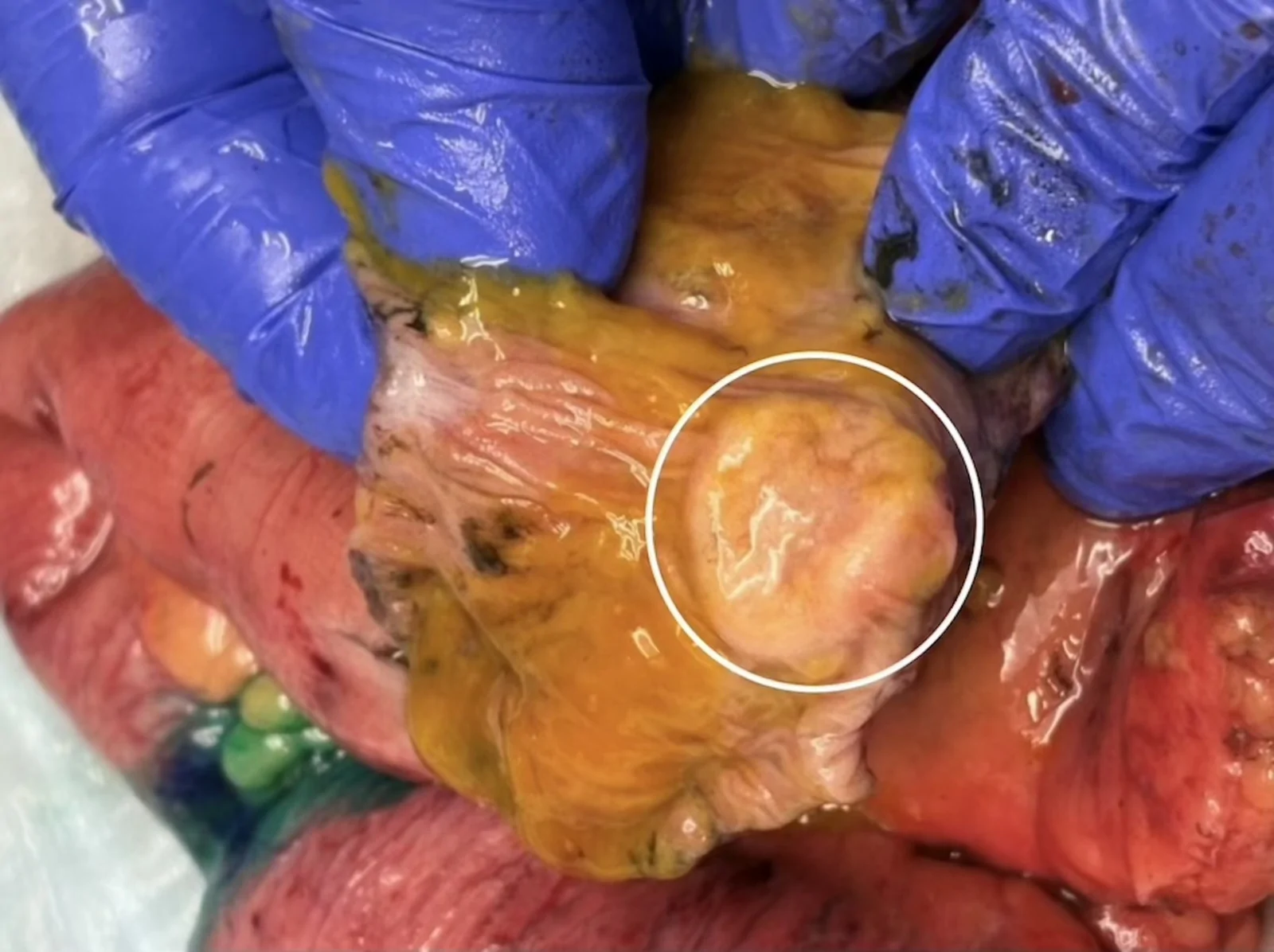
Luminal small bowel mass The white circle demonstrates an intraluminal mass.

**Figure 5:**
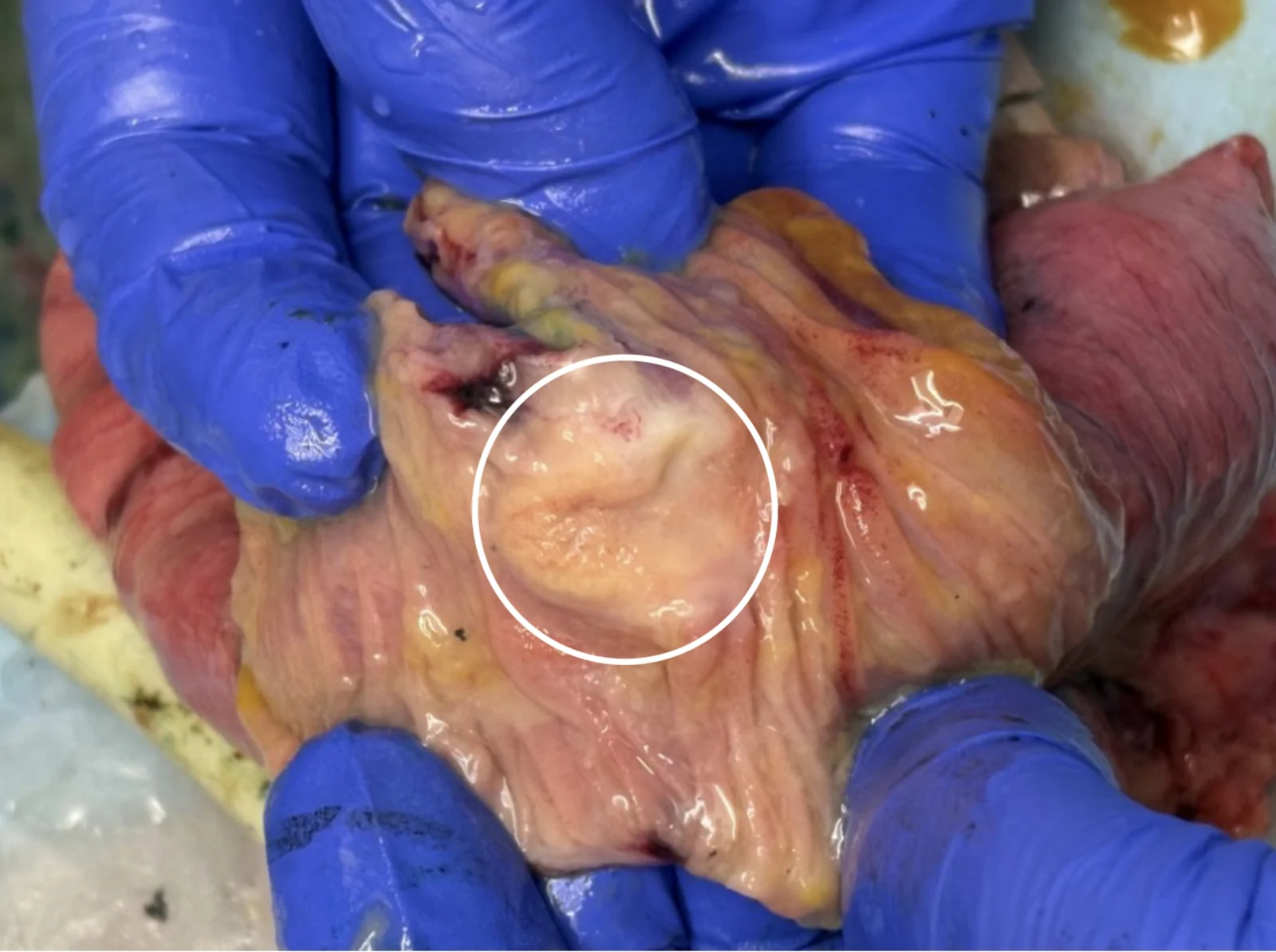
Another image demonstrating intraluminal mass A white circle indicates an intraluminal mass.

The differential diagnosis included infectious as well as malignant causes, including metastatic lymphadenopathy and sarcoma. The patient was taken to the operating room for robot-assisted diagnostic laparoscopy with possible biopsy of the mesenteric mass. Exploration of the abdomen revealed two small bowel luminal masses, likely malignant, in close proximity to the mesenteric mass. The two small bowel masses, along with the mesenteric mass, were resected, and the bowel was primarily anastomosed. The gross specimen can be seen in Figures 3-5. 

**Figure 6:**
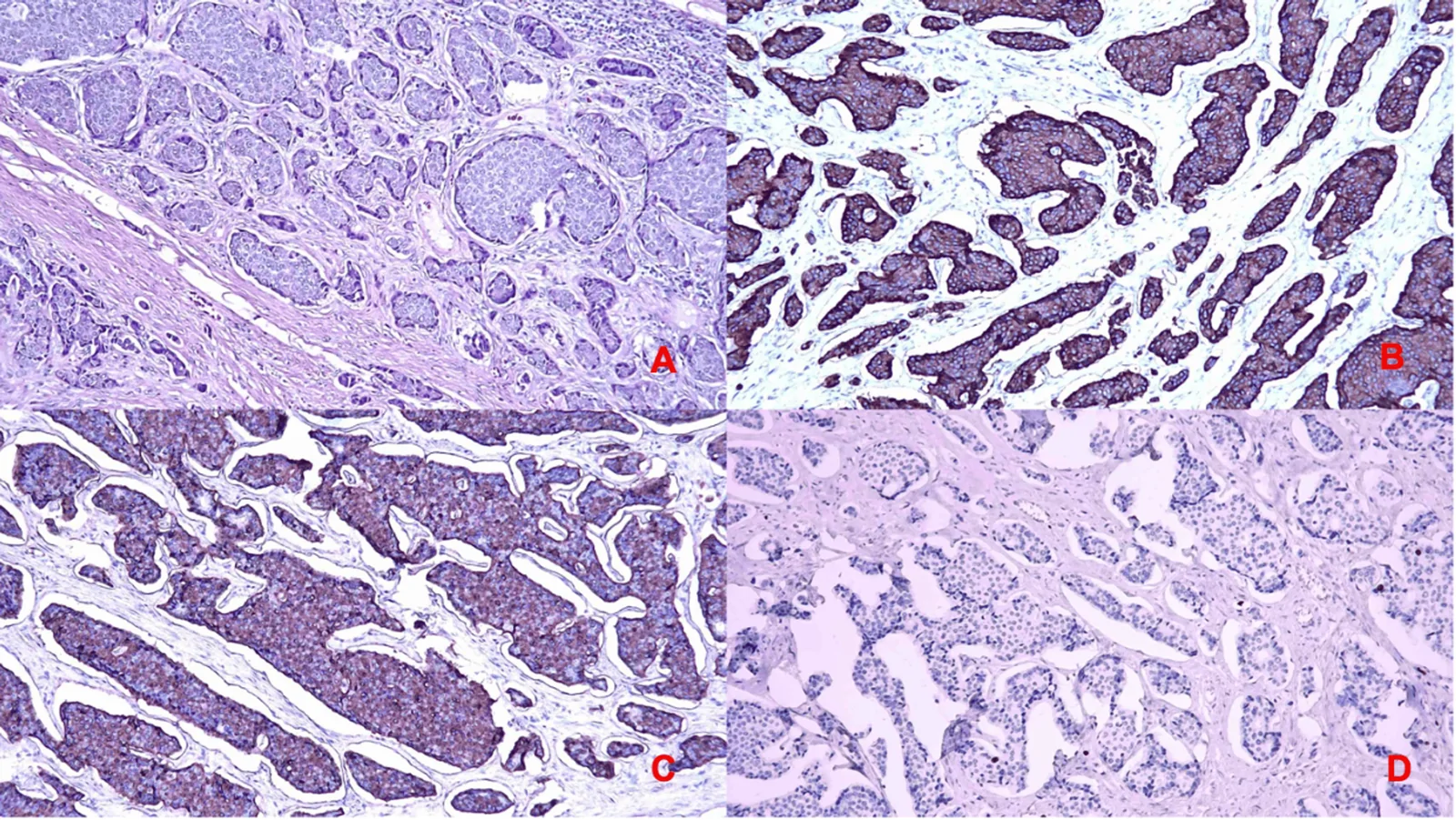
Low-grade neuroendocrine neoplasm in the jejunum: The neoplastic cells demonstrate the organoid growth pattern with various sizes of small nodules (A). The neoplastic cells are immunoreactive to AE1/AE3, NSE, synaptophysin (B), and chromogranin (C) with a 1-2% proliferative rate stained by Ki67 (D). These features are consistent with low-grade neuroendocrine neoplasm. The image is at 100x magnification

**Figure 7:**
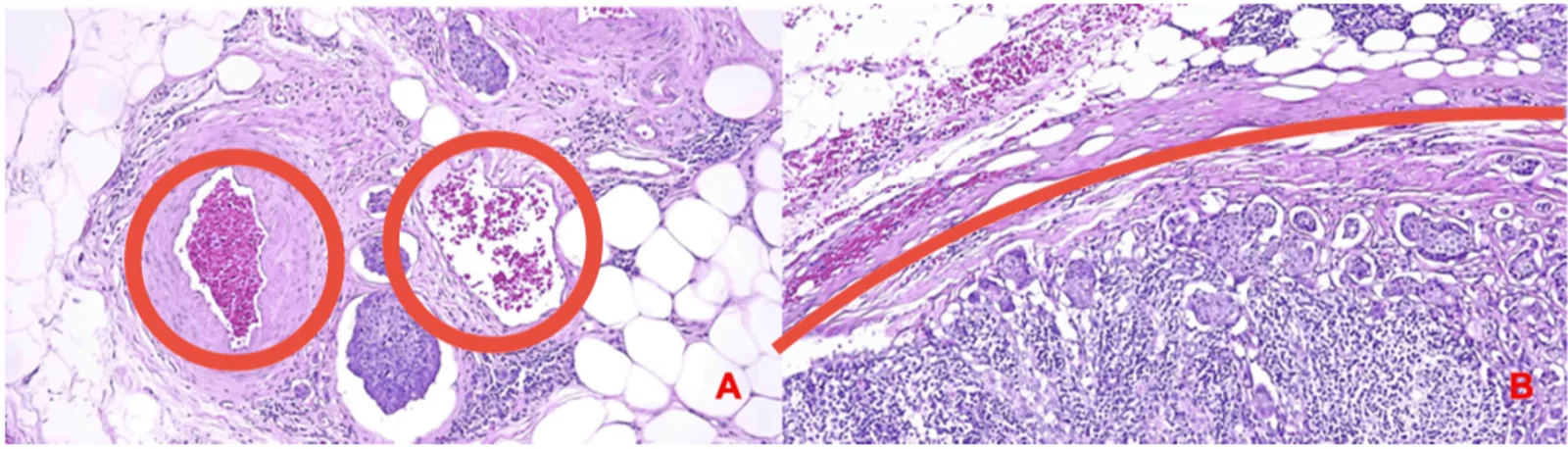
Low-grade neuroendocrine neoplasm in the jejunum: The lymphovascular invasion (circled in red on image A) with extensive lymph node metastasis (B) is present. Stained by Ki67. The image is at 100x magnification

The postoperative course was uneventful, with no evidence of immediate complications. Computed tomography of the chest revealed no evidence of metastatic disease. The patient was subsequently discharged on the second postoperative day in stable condition. Pathology reports for said mass resulted in a low-grade neuroendocrine neoplasm of the small bowel, demonstrated in Figures 6, 7. 

This patient’s case was discussed in a multidisciplinary tumor board at Brookdale University Medical Center of Brooklyn, NY, and is currently being followed up in regard to the necessity for chemotherapy.

## Discussion

As mentioned in the study by Gagliardi et al. [1], small bowel neuroendocrine tumors (SBNETs) are the most common primary malignancy of the small intestine, with a rising incidence attributed to improved imaging, endoscopic techniques, and increased clinical awareness. These tumors typically arise from enterochromaffin cells and are often indolent but can exhibit aggressive behavior, particularly in high-grade cases, although this was not observed in the present case. The majority of SBNETs are sporadic, though familial forms and associations with hereditary syndromes such as MEN1 and MEN2 have been described [9-10,15].

An important feature of this patient’s presentation is the complete absence of symptoms, which is increasingly recognized in SBNETs due to incidental detection on imaging performed for unrelated reasons. Most SBNETs remain clinically silent until advanced disease, with symptoms such as abdominal pain, obstruction, or carcinoid syndrome typically manifesting only with significant tumor burden or metastatic spread, which coincides with this patient's low-grade presentation on pathology. Asymptomatic patients may also harbor advanced disease, and the decision to pursue surgical intervention in this population remains controversial [15,16]. The tumor in this case was located in the small bowel, a site where NETs are most frequently multifocal and may be sub-centimeter in size, necessitating careful intraoperative palpation of the entire bowel to identify synchronous lesions. Multifocality is present in up to 44% of cases, and missed lesions can lead to recurrence or persistent symptoms. The absence of cystic features in this mass is consistent with the majority of SBNETs, which are solid and may be associated with cystic features, desmoplastic reaction, or mesenteric fibrosis, particularly in cases with nodal metastases [1-4,6,17].

It is important to discuss high-grade SBNETs, which are less common but portend a more aggressive clinical course, with an increased risk of nodal and distant metastases, rapid progression, and poorer prognosis. Histologic grade, determined by mitotic rate and Ki-67 index, is a key determinant of management and outcome, with poorly differentiated tumors requiring more aggressive multimodal therapy [3,5,10,12]. Imaging modalities such as multiphasic CT, MRI, and somatostatin receptor-based PET/CT are essential for staging and guiding treatment, especially in high-grade disease [15,17]. Surgical resection remains the cornerstone of management for localized and select metastatic SBNETs, with complete excision of the primary tumor and regional lymphadenectomy recommended to prevent recurrence and alleviate complications. Intraoperative assessment should include manual palpation of the entire bowel and evaluation of proximity to the superior mesenteric vessels, as mesenteric involvement can complicate resection and increase the risk of short bowel syndrome. Extended lymphadenectomy is associated with improved survival, and the lymph node ratio is emerging as a prognostic marker [1-8,17]. The role of surgery in asymptomatic, high-grade, or metastatic SBNETs is debated. Recent cohort studies suggest that non-operative management in asymptomatic stage IV patients is associated with a low incidence of symptom development and limited progression of mesenteric metastases, though some guidelines advocate for prophylactic resection to prevent future complications. Individualized decision-making, incorporating patient comorbidities, tumor burden, and multidisciplinary input, is essential [2,4-7,9-11,14-17].

Minimally invasive surgical techniques, including laparoscopic and robotic approaches, are increasingly utilized for SBNETs, offering reduced postoperative pain and faster recovery. However, open surgery remains the gold standard for multifocal or high-grade disease due to the need for thorough palpation and complex lymphadenectomy. The choice of approach should be guided by preoperative imaging, intraoperative findings, and surgeon expertise, with a low threshold for conversion to open surgery if oncologic or technical goals cannot be achieved [1, 7-8,17].

Adjuvant and systemic therapies play a critical role in the management of high-grade and metastatic SBNETs. Somatostatin analogues, peptide receptor radionuclide therapy, targeted agents, and chemotherapy are utilized based on tumor grade, functional status, and extent of disease. Multimodal therapy improves symptom control and progression-free survival, particularly in advanced cases [5,10,14,15,17].

Long-term outcomes for SBNETs are generally favorable, with five-year survival rates exceeding 70% in population-based studies, though prognosis is influenced by stage, grade, and the presence of mesenteric tumor deposits. Ongoing surveillance post-resection is recommended, with periodic imaging and biochemical assessment to detect recurrence or progression. The increasing incidence and complexity of SBNETs underscore the importance of multidisciplinary management, early diagnosis, and individualized treatment strategies to optimize patient outcomes [1-17].

## Conclusions

This case highlights the evolving understanding of small bowel neuroendocrine tumors (SBNETs), demonstrating how advances in imaging and minimally invasive surgery have improved the detection of lesions that often remain clinically silent until advanced stages. The incidental identification of multifocal, low-grade disease in an asymptomatic patient, in turn, does not exclude the possibility of significant tumor burden as reviewed in the adjacent literature and underscores the importance of maintaining a broad differential when evaluating mesenteric or small bowel masses. Furthermore, the frequent multifocality of SBNETs necessitates meticulous intraoperative assessment, including complete exploration and palpation of the small intestine, to ensure accurate staging and optimal oncologic resection while preserving bowel length and vascular integrity.

Pathologic grade remains a key determinant of prognosis and guides the need for adjunctive therapy, particularly in high-grade tumors with increased risk of progression and metastasis. As reflected in both this case and the broader literature, optimal management of SBNETs relies on a multidisciplinary approach integrating surgery, systemic therapy, and longitudinal surveillance. Continued advances in imaging, biomarkers, and targeted treatments will further refine risk stratification and individualized care. Ultimately, early recognition, comprehensive surgical management, and coordinated follow-up are essential to improving outcomes for patients with these increasingly encountered neoplasms.
